# Comparative N-Glycoproteomics Analysis of Clinical Samples Via Different Mass Spectrometry Dissociation Methods

**DOI:** 10.3389/fchem.2022.839470

**Published:** 2022-02-24

**Authors:** Wenjuan Zeng, Shanshan Zheng, Tao Su, Jiahan Cheng, Yonghong Mao, Yi Zhong, Yueqiu Liu, Jianhai Chen, Wanjun Zhao, Tianhai Lin, Fang Liu, Guisen Li, Hao Yang, Yong Zhang

**Affiliations:** ^1^ Institutes for Systems Genetics, National Health Commission (NHC) Key Laboratory of Transplant Engineering and Immunology, West China Hospital, Sichuan University, Chengdu, China; ^2^ Department of Thoracic Surgery, Institute of Thoracic Oncology, West China Hospital, Sichuan University, Chengdu, China; ^3^ Department of Thyroid Surgery, West China Hospital, Sichuan University, Chengdu, China; ^4^ Department of Urology, Institute of Urology, West China Hospital, Sichuan University, Chengdu, China; ^5^ Division of Nephrology, West China Hospital, Sichuan University, Chengdu, China; ^6^ Renal Department and Institute of Nephrology, Sichuan Provincial People’s Hospital, University of Electronic Science and Technology of China, Sichuan Clinical Research Center for Kidney Diseases, Chengdu, China

**Keywords:** N-glycosylation, clinical sample, glycoproteomics, mass spectrometry, electron-transfer/higher-energy collisional dissociation (EThcD)-stepped collision energy/higher-energy collisional dissociation (sceHCD)

## Abstract

Site-specific N-glycosylation characterization requires intact N-glycopeptide analysis based on suitable tandem mass spectrometry (MS/MS) method. Electron-transfer/higher-energy collisional dissociation (EThcD), stepped collision energy/higher-energy collisional dissociation (sceHCD), higher-energy collisional dissociation-product-dependent electron-transfer dissociation (HCD-pd-ETD), and a hybrid mass spectrometry fragmentation method EThcD-sceHCD have emerged as valuable approaches for glycoprotein analysis. However, each of them incurs some compromise, necessitating the systematic performance comparisons when applied to the analysis of complex clinical samples (e.g., plasma, urine, cells, and tissues). Herein, we compared the performance of EThcD-sceHCD with those previous approaches (EThcD, sceHCD, HCD-pd-ETD, and sceHCD-pd-ETD) in the intact N-glycopeptide analysis, and determined its applicability for clinical N-glycoproteomic study. The intact N-glycopeptides of distinct samples, namely, plasma from prostate cancer (PCa) patients, urine from immunoglobulin A nephropathy (IgAN) patients, human hepatocarcinoma cell line (HepG2), and thyroid tissues from thyroid cancer (TC) patients were analyzed by these methods. We found that EThcD-sceHCD outperformed other methods in the balance of depth and accuracy of intact N-glycopeptide identification, and sceHCD and EThcD-sceHCD have good complementarity. EThcD-sceHCD holds great potential for biomarker discovery from clinical samples.

## Introduction

Glycosylation has been recognized as the most prevalent, structurally diverse, and multifunctional posttranslational modification of proteins (Moremen et al., 2012). It plays vital roles in physiopathological processes, including the progression of various cancers (Ohtsubo and Marth, 2006). Glycoproteomic (glycoprotein, glycosite, glycan, and intact glycopeptide) analysis of clinical samples can reveal tumor biomarkers and therapeutic targets (Zhang et al., 2020a; Cao et al., 2021). Among them, intact glycopeptide analysis can simultaneously obtain glycoprotein, glycosite, and glycan composition information. Hence, it has become the major method in the glycoproteomics study. However, the intact glycopeptide analysis is very challenging owing to the macroheterogeneity and microheterogeneity of glycosylation modification, low stoichiometry of intact glycopeptides, and different energy requirements to fragment the peptides and glycans (Pujić and Perreault, 2021). How to achieve the accurate and in-depth identification of intact glycopeptides is a critical problem in the clinical glycoproteomic study.

Over the past decades, a lot of researchers have come up with their own solutions. Efforts are mainly made in the following three areas: sample preparation, mass spectrometry analysis, and data processing. In sample preparation, many glycoproteins or intact glycopeptide enrichment materials or methods [e.g., hydrophilic interaction chromatography (HILIC), titanium dioxide chromatography, lectin affinity chromatography, boronate affinity chromatography, and hydrazide chemistry] were developed to avoid the interference of nonglycoprotein or nonglycopeptides (Palmisano et al., 2010; Sun et al., 2017; Yang et al., 2017; Zhang et al., 2017; Wu et al., 2018; Chen et al., 2019; Sun et al., 2019; Zhang et al., 2019; Zhang et al., 2020b). Besides, protein extraction and digestion will also affect the accuracy and depth of intact glycopeptide identification. In terms of mass spectrometry, varied fragmentation techniques for tandem mass spectrometry (MS/MS) analysis, such as electron-transfer/higher-energy collisional dissociation (EThcD), stepped collision energy/higher-energy collisional dissociation (sceHCD), higher-energy collisional dissociation-product-dependent electron-transfer dissociation (HCD-pd-ETD), have emerged as valuable approaches for clinical glycoproteomics (Saba et al., 2012; Ma et al., 2016; Yu et al., 2017). Yu’s team and Zhang’s team proved that EThcD is a powerful and effective tool for the analysis of intact N-glycopeptides and intact O-glycopeptides of human plasma in that EThcD produces glycan-attached c/z ions for the identification of both peptide backbone and glycosites as well as the glycan compositions (Yu et al., 2017; Zhang et al., 2018). However, each of these collision modes has advantages and disadvantages (Riley et al., 2020). In terms of data processing, the annotation of intact glycopeptide MS/MS data is very challenging, including the correct assignment of glycan composition, glycosite as well as the peptide backbone (Kawahara et al., 2021). Recently, many intact glycopeptides search engines have been developed based on modern MS techniques. These tools, especially Byonic, MSFragger-Glyco, pGlyco, StrucGP, O-pair, have gradually improved the identification depth or accuracy for glycoproteomics (Bern et al., 2012; Liu et al., 2017; Lu et al., 2020; Polasky et al., 2020; Shen et al., 2021). Facilitated by the HUPO Human Glycoproteomics Initiative (HGI), Kawahara et al. performed a comprehensive community-based evaluation of existing informatics solutions for large-scale intact glycopeptide analysis. Their study involved several high-performance search strategies and specified key variables that will help informatics decision making in glycoproteomics (Kawahara et al., 2021).

In this study, we focused on improving the depth and accuracy of intact glycopeptide identification from complex samples based on the advanced mass spectrometry analysis. In a recent work, we have presented a hybrid dissociation technique, EThcD-sceHCD, by integrating EThcD and sceHCD into a sequential glycoproteomic workflow (Zhang et al., 2021a; Zhang et al., 2021b). Our results have indicated that EThcD-sceHCD is able to achieve improved performance in the analysis of intact N/O-glycopeptides of a highly glycosylated protein (HIV-1 gp120) compared with other methods. Furthermore, we analyzed purified IgG subclasses (IgG1, IgG2, IgG3, and IgG4) from patients with chronic kidney disease using EThcD-sceHCD (Zhang et al., 2021a; Zhang et al., 2021b). When compared with EThcD and sceHCD, EThcD-sceHCD obtained more informative fragment ions, higher spectral quality, higher Byonic score, and more intact glycopeptide identifications. Does this approach also work well for complex clinical samples (such as plasma, urine, cells, and tissues)? Herein, we compared EThcD-sceHCD with previously reported methods, sceHCD, EThcD, HCD-pd-ETD, and sceHCD-pd-ETD, in the analysis of intact N-glycopeptides from complex clinical samples for the first time, and determined its applicability for clinical N-glycoproteomic study. The raw MS data have been deposited to the ProteomeXchange Consortium *via* the PRIDE partner repository with the dataset identifier *PXD030288*.

## Experimental section

### Materials and chemicals

Dithiothreitol (DTT), iodoacetamide (IAA), sodium dodecyl sulfate (SDS), and ammonium bicarbonate (ABC) were obtained from Sigma-Aldrich (St. Louis, MO, USA). Protease inhibitor cocktail tablets were purchased from Roche Diagnostics (Sandhofer Strasse, Mannheim, Germany). Sequencing-grade trypsin and Lys-C were obtained from Promega (Madison, WI, USA). Trifluoroacetic acid (TFA), acetonitrile (ACN), and formic acid (FA) were purchased from Thermo Fisher Scientific (Waltham, MA, USA). Water used in the experiments was purified by a Milli-Q system (Millipore, Bedford, MA, United States). Zwitterionic HILIC (Zic-HILIC) was purchased from Fresh Bioscience (Shanghai, China). The C8 extraction disks were purchased from 3M Empore (St. Paul, MN, USA). Sterile cell culture plates were purchased from Corning (New York, NY, USA). Dulbecco’s modified Eagle medium (DMEM), fetal bovine serum (FBS), penicillin–streptomycin (PS) antibiotics, phosphate-buffered saline (PBS), and trypsin were obtained from Gibco (Waltham, MA, USA). All other chemicals and reagents of the best available grade were obtained from Sigma-Aldrich or Thermo Fisher Scientific.

### Clinical sample collection

Diagnosis and pathological analyses of prostatic cancer (PCa) were performed in the Department of Urology, West China Hospital. Diagnosis and pathological analyses of thyroid cancer (TC) were performed in the Department of Thyroid Surgery, West China Hospital. Diagnosis and pathological analyses of Immunoglobulin A nephropathy (IgAN) were performed in the Renal Department and Institute of Nephrology, Sichuan Provincial People’s Hospital. All patients underwent tissue biopsy and were histopathologically diagnosed according to the criteria of the World Health Organization. Blood from PCa patients was collected in an ethylenediaminetetraacetic acid anticoagulant tube and centrifuged at 300 × *g* for 10 min at 4°C. Plasma was collected in 1-ml tubes and stored at –80°C until use. The midstream urine from IgAN patients was collected in an appropriate container and centrifuged at 1,000 × *g* for 10 min. The precipitate was discarded, and 10 ml of the supernatant was collected in a 50-ml tube and stored at −80°C until use. The thyroid tissue from thyroid cancer patients was collected within 30 min after the operation, cleaned with a sterile towel, transferred to sterile freezing vials, immersed in liquid nitrogen, and stored at −80°C until use. All experiments were performed following the guidelines of Chinese Medical Ethics Committee, and approved by the ethics committee at Sichuan Provincial People’s Hospital and West China Hospital. Informed consent was obtained for any experimentation with human subjects.

### Cell culture

Human hepatocarcinoma cell line (HepG2) was obtained from the American Type Culture Collection (ATCC) and authenticated through Short Tandem Repeat (STR) profiling. HepG2 cells were cultured in DMEM supplemented with 5% FBS and 1% PS, and grown at 5% CO_2_ in a humidified incubator at 37°C.

### Protein extraction

Cells were harvested when reaching 80% confluence and washed with ice-cold PBS three times. Then the cells were lysed by UA buffer (8 M urea, 0.1 M Tris, pH 8.5, 1% protease inhibitor cocktail) with sonication (SCIENTZ-IID, Ningbo Scientz Biotechnology Co., Ltd., Ningbo, China) at 4°C for protein extraction. The lysate was centrifuged at 13,000 × *g* for 20 min at 4°C. The supernatant was transferred to a clean tube and stored at −80°C. Approximately 80 mg of thyroid cancer tissues were homogenized in lysis buffer (8 M urea, 50 mM Tris, pH 8.5, 1% protease inhibitor cocktail) using a tissue lyser (speed = 6.5 m/s; time = 60 s; number of cycles = 3; Lifereal Biotechnology Co., Ltd., Hangzhou, China) followed by sonication at 4°C. The homogenate was centrifuged at 13,000 × *g* for 20 min at 4°C. The supernatant was then collected and stored at −80°C until use. Plasma was subpacked and stored at −80°C until use. Urine supernatant was concentrated using a 30-kDa ultrafiltration tube (Millipore) and stored at −80°C until use. Concentration of proteins extracted from cells, tissues, plasma, and urine was determined by Bradford assay (Thermo Fisher Scientific).

### Reduction, alkylation, and digestion

Proteins extracted from plasma from PCa patients, urine from IgAN patients, HepG2 cells, and thyroid cancer tissues were processed and digested following the filter-aided sample preparation (FASP) protocol. Briefly, 500 µg of proteins was diluted with UA buffer and loaded onto a 30-kDa filter tube. After being centrifuged at 13,000 × *g* for 15 min at 25°C, the proteins were reduced by 20 mM DTT for 4 h at 37°C followed by alkylation with 50 mM IAA for 30 min at 25°C in the dark. The protein mixture was washed twice with 200 μl of UA buffer and four times with 200 μl of 50 mM ABC by centrifugation at 13,000 × *g* for 15 min at room temperature. Then 200 μl of ABC containing 10 μg of trypsin and 10 μg of Lys-C were added to each filter tube and incubated for 16 h at 37°C. Finally, the filter tubes were washed three times with 100 μl of water, and the flow-through fractions containing peptides were collected. The peptide concentration was determined using a quantitative colorimetric peptide assay kit (Thermo Fisher Scientific) based on the absorbance at 480 nm. The peptide mixtures were freeze dried and then stored at −80°C.

### Intact N-glycopeptide enrichment

Intact N*-*glycopeptides were enriched by Zic-HILIC materials. Specifically, 10 mg of Zic-HILIC beads were washed three times for 10 min with 0.1% TFA and 70% ACN/0.2% TFA. Then 200 μg of tryptic peptides were dissolved in 70% ACN/0.2% TFA solution, added into the processed Zic-HILIC beads and rotated for 2 h at 37°C to enrich the intact N-glycopeptides. Last, the mixture was transferred to a 200-μl pipet tip packed with a layer of C8 membrane and washed twice with 70% ACN/0.2% TFA. Intact N*-*glycopeptides bound on the Zic-HILIC beads were eluted three times with 70 μl of 0.1% TFA. The pooled eluent was dried under vacuum for subsequent LC-MS/MS analysis.

### LC-MS/MS analysis

The intact N-glycopeptides derived from plasma, urine, cells, and tissues were dissolved with 40 μl of loading buffer (0.1% FA in water), respectively. Peptide solution (2 μl) was loaded for LC-MS/MS analysis using an Orbitrap Fusion Lumos mass spectrometer (Thermo Fisher Scientific) with the Xcalibur software (version 4.3; Thermo Fisher Scientific). The intact N-glycopeptides were separated on a C18 column (ReproSil-Pur C18-AQ, 1.9 μm, 75 μm × 20 cm; Dr. Maisch) at a flow rate of 300 nl/min over a 78-min gradient (solvent A, 0.1% FA in water; solvent B, 0.1% FA in 80% ACN; 0–8 min, 5%–8% B; 8–58 min, 8%–22% B; 58–70 min, 22%–32% B; 70–71 min, 32%–90% B; and 71–78 min, 90% B), and detected in the data-dependent acquisition mode. Notably, five different MS/MS dissociation modes, EThcD, sceHCD, EThcD-sceHCD, HCD-pd-ETD, and sceHCD-pd-ETD were used for the fragmentation of intact N-glycopeptides. Each sample was detected in three technical replicates for every MS/MS dissociation method. The detailed parameters for the five dissociation modes were as follows.

The MS1 scan range, RF lens, exclusion duration, and MS2 first mass were set as 800–2,000 (*m/z*), 40%, 15 s, and 120 (*m/z*) in all the five modes. The MS1 and MS2 were acquired at an Orbitrap resolution of 60,000 and 30,000, respectively, in all the five methods except for sceHCD-MS/MS, where the Orbitrap resolution was set as 120,000 for MS1 acquisition and 15,000 for MS2 analysis. The precursor ion for MS2 analysis was isolated with an isolation window of 2.0 (*m/z*) in all methods except for the sceHCD cycle of EThcD-sceHCD-MS/MS, which used an isolation window of 1.6 (*m/z*).

For EThcD-MS/MS, the maximum injection time and AGC target were set as 50 ms and custom (2e^5^) in MS1, and 150 ms and custom (5e^5^) in MS2. EThcD normalized collision energy was 35%, and the cycle time was 3 s.

For sceHCD-MS/MS, the maximum injection time and AGC target were 100 ms and custom (2e^5^) in MS1, and 250 ms and custom (5e^5^) in MS2. Stepped collision energy HCD mode was turned on with an energy difference of ±10% (20%–30%–40%). The cycle time was 3 s.

For EThcD-sceHCD-MS/MS, the data acquisition for the EThcD-sceHCD-MS/MS was performed using an alternative fragmentation between EThcD and sceHCD modes in a duty cycle. In duty cycle 1 (EThcD), the maximum injection time and AGC target were 50 ms and custom (2e^5^) for MS1, and 150 ms and custom (5e^5^) in MS2. The EThcD normalized collision energy was set as 35%. The EThcD cycle time was 2 s. In duty cycle 2 (sceHCD), the maximum injection time and AGC target were auto and standard in MS1, and auto and custom (1e^5^) in MS2. Stepped collision mode was turned on with HCD normalized collision energies set as 20%–30%–40%. The sceHCD cycle time was 1 s.

For HCD-pd-ETD-MS/MS or sceHCD-pd-ETD-MS/MS, the product-dependent methods were constructed using two classic dissociation types as the triggered scan. AGC target and maximum injection time were custom (2e^5^) and 50 ms in MS1. Product-dependent triggering required at least one ion from the following list to be present in the top 20 most abundant peaks in a spectrum within 15 ppm tolerance: 138.0545, 204.0867, and 366.1396 (*m/z*). AGC target and maximum injection time were custom (5e^5^) and 150 ms in MS2. The ETD mode was turned on with an energy of 35%. Stepped collision energy mode (for sceHCD-pd-ETD) was turned on with an energy difference of ±10% (20%–30%–40%). The cycle time was 3 s.

### Data analysis

The MS raw data files of intact N*-*glycopeptides were searched against the human Uniprot database (version 2015_03, 20,410 entries) using the Byonic software (version 3.6.0, Protein Metrics, Inc.). The mass tolerance for precursors and fragment ions were set as ±10 and ±20 ppm, respectively. The fixed modification was carbamidomethylation of cysteine, and variable modifications included oxidation of methionine and acetylation of protein N-terminal. Two missed cleavage sites were allowed for enzyme digestion. All other parameters were set at the default values. In addition, the 182 human N-glycans were specified as the N-glycosylation modification for all searches. Protein groups were filtered to 1% false discovery rates (FDRs) based on the number of hits obtained for searches against these databases. Stricter quality control criteria for intact N-glycopeptide identification were used, including a Byonic score over 200 and identification of at least seven amino acids.

### Statistical analysis

Analysis of variance (ANOVA) was applied to the statistical comparison among five groups in the number of intact N-glycopeptides, N-glycan compositions, and N-glycoproteins identified based on different fragmentation modes for each sample. Student’s t-test was used for the binary statistical comparison. Data were shown as means ± standard deviation (SD), and a *p*-value <0.05 was considered significant.

## Results and discussion

To characterize the site-specific N-glycosylation of proteins from complex clinical samples, the choice of suitable fragmentation mode is the key. Generally, HCD, the representative of beam-type collisional activation, and ETD, the typical electron-based dissociation, are two fundamental and commonly used MS/MS fragmentation methods for glycopeptide characterization. Because of their distinct activation mechanisms, HCD and ETD produce different fragments when applied to the dissociation of intact glycopeptides. HCD generates b/y-type fragments derived from the peptide backbone that lose part or all of the modified glycans due to the fragmentation of glycans by HCD, while ETD yields mainly c/z-type peptide backbone fragments retaining the intact glycan moieties (Riley et al., 2020). Hence, they share good complementarity to each other.

Based on the above two basic approaches, various fragmentation methods have been developed, including the optimized HCD (e.g., sceHCD) and the paired HCD and ETD (e.g., EThcD, HCD-pd-ETD, and sceHCD-pd-ETD) (Saba et al., 2012; Yu et al., 2017). Taking advantage of the stepped collision energies, sceHCD can provide better fragmentation for both glycans and peptide backbone as lower collision energies have been found to benefit glycan fragmentation, while higher collision energies aid in better dissociation of peptide backbone. Accordingly, previous reports suggest that sceHCD-MS/MS can generate the most abundant and informative fragment ions from both the peptide backbone and the attached glycan of an intact N-glycopeptide in one single spectrum compared with CID-, HCD-, ETD-, ETciD- and EThcD-MS/MS (Klein and Zaia, 2020; Riley et al., 2020). However, sceHCD-MS/MS cannot provide sufficient spectral evidence for accurate location of N/O-glycosite and identification of glycan composition in the case when more than one glycosite occurs within one peptide (Zhang et al., 2021b). EThcD-, HCD-pd-ETD-, and sceHCD-pd-ETD-MS/MS can produce a more complete fragmentation and a greater proportion of fragment ions of glycopeptides because the precursor ions are fragmented *via* both ETD and HCD, thereby enabling the glycosites and glycans to be unambiguously determined (Saba et al., 2012; Ma et al., 2016; Yu et al., 2017).

Unfortunately, the dissociation efficiency of these methods is still limited, especially for the glycopeptide precursors with low charge density. Hence, we proposed a combined fragmentation mode, EThcD-sceHCD-MS/MS, which was implemented using an alternative fragmentation between EThcD and sceHCD mode in a duty cycle (Zhang et al., 2021b). This combined fragmentation strategy makes good use of both sceHCD and EThcD with higher dissociation efficiency and better spectra quality. Our previous studies have shown that EThcD-sceHCD can achieve improved performance for the analysis of intact glycopeptides from highly glycosylated proteins, HIV-1 gp120 and IgG subclasses (Zhang et al., 2021a; Zhang et al., 2021b). Herein, we applied this method to the analysis of intact N-glycopeptides from varying types of clinical samples and systematically compared with several other methods that are commonly used in glycoproteomics (i.e., sceHCD, EThcD, HCD-pd-ETD, and sceHCD-pd-ETD) to evaluate their performance in characterizing intact N-glycopeptides from the complex clinical samples.

**FIGURE 1:**
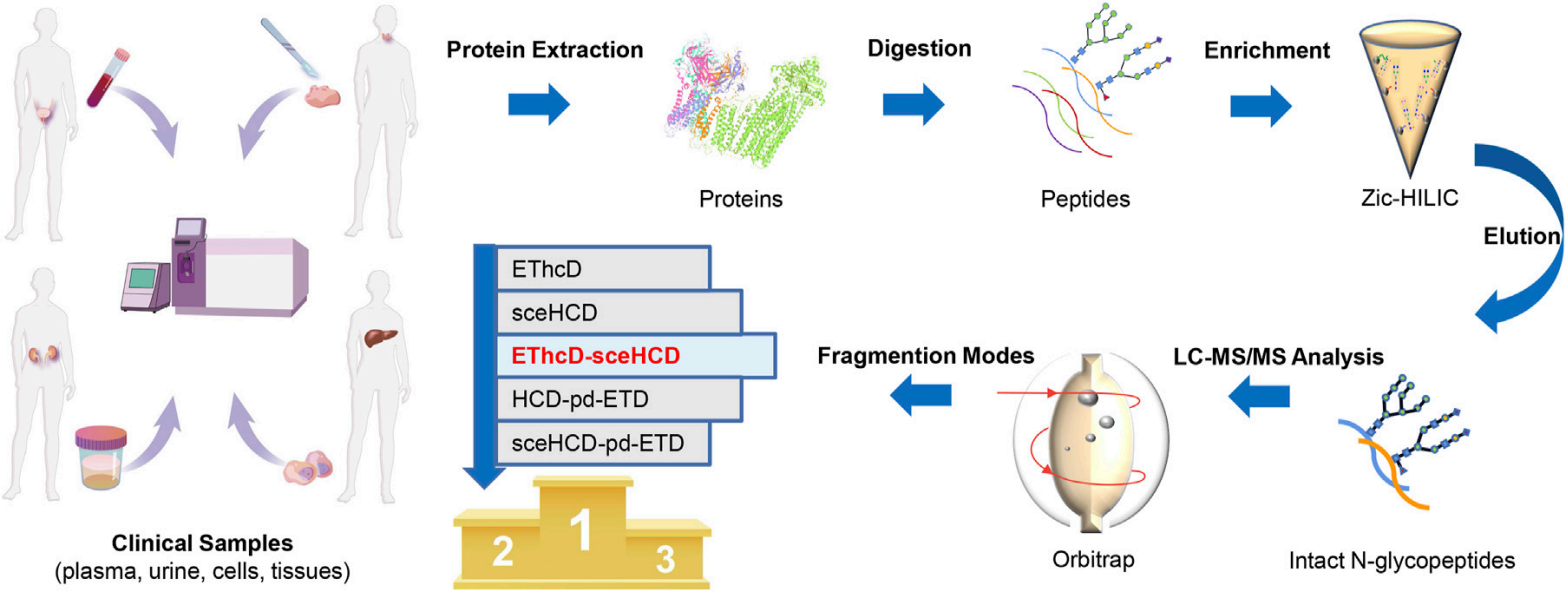
Schematic illustration of the workflow for human plasma, urine, cells, and tissue intact N-glycopeptides analysis using different dissociation methods.

To evaluate the usefulness and superiority of the EThcD-sceHCD method in complicated clinical samples, the following experiments were designed (Figure 1). Specifically, proteins extracted from plasma from PCa patients, urine from IgAN patients, HepG2 cells, and thyroid cancer tissues were digested by trypsin and Lys-C. The obtained peptides were enriched with Zic-HILIC materials. Then the enriched intact N-glycopeptides from plasma, urine, cells, and tissues were analyzed three times repeatedly using different fragmentation modes (i.e., EThcD, sceHCD, EThcD-sceHCD, HCD-pd-ETD, and sceHCD-pd-ETD) (Figure 1). All the MS raw data were searched by the Byonic software, and for all methods, the localized N-glycopeptide spectral matches (N-glycoPSMs) were filtered to get a Byonic score of over 200 and a peptide length of at least seven amino acids to ensure quality identifications. Statistical analysis was performed to systematically compare the number of identified N-glycoPSMs, N-glycans, intact N-glycopeptides, and N-glycoproteins among the five fragmentation modes (Supplementary Tables S1–S4).

**FIGURE 2:**
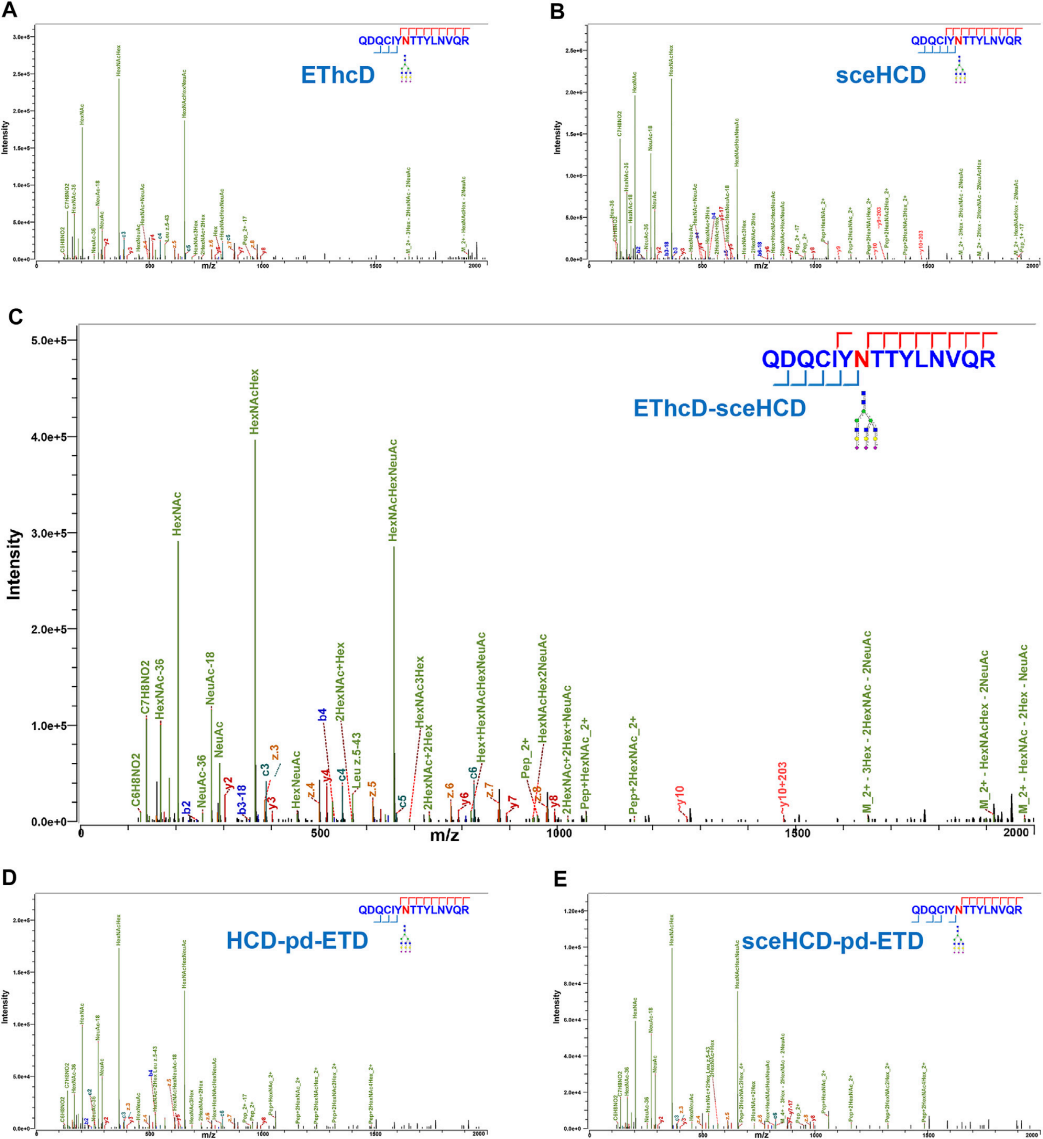
Comparison of electron-transfer/higher-energy collisional dissociation (EThcD), stepped collision energy/higher-energy collisional dissociation (sceHCD), EThcD-sceHCD, higher-energy collisional dissociation-product-dependent electron-transfer dissociation (HCD-pd-ETD), and sceHCD-pd-ETD spectra of alpha-1-acid glycoprotein 1 N-linked glycopeptide (N93) from human plasma.

In general, the five fragmentation methods produce abundant fragment ions with distinct features. For example, as shown in Figure 2, all methods can provide confident N-glycosite (N93) localization and abundant information about the N-glycan composition [HexNAc(5)Hex(6)NeuAc(3)] of an intact N-glycopeptide from human plasma alpha-1-acid glycoprotein 1. For EThcD-, EThcD-sceHCD-, HCD-pd-ETD-, and sceHCD-pd-ETD-MS/MS, the precursor ions or fragment ions were dissociated with both ETD and HCD. Specifically, the product-dependent mode was used to trigger a scan of specific ETD to fragment the glycopeptide upon the detection of glycopeptide-specific oxonium ions produced by HCD (Yin et al., 2013). Their spectra included glycan fragments, b/y/c/z-type peptide backbone fragments, and Y ions (y-type peptide backbone fragments attached by glycan) with few occurrences of glycan dissociation (Figures 2A,C–E). sceHCD-MS/MS spectrum contained b/y-type peptide backbone fragments without glycans, oxonium ions, and Y ions (Figure 2B).

**FIGURE 3:**
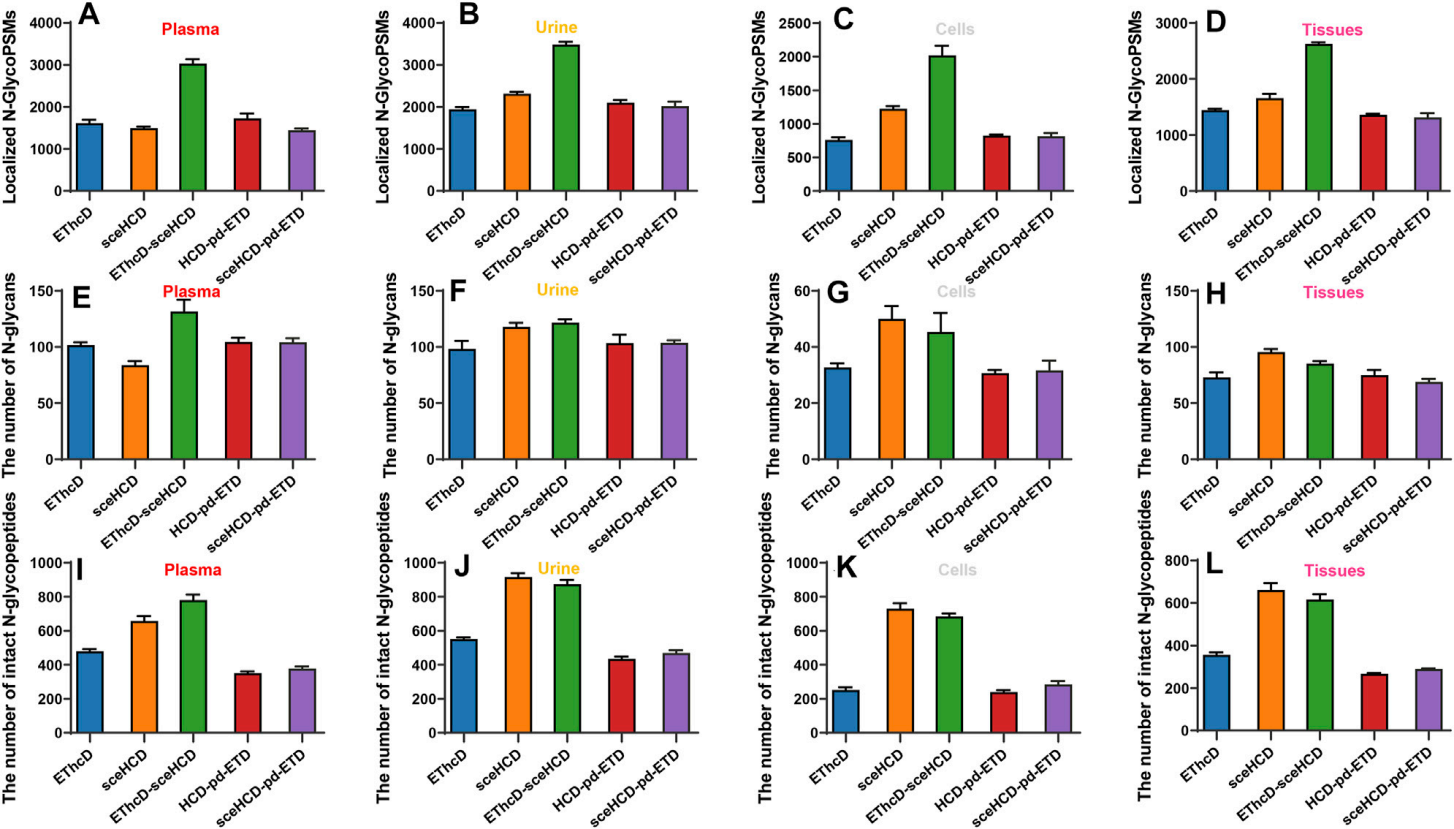
Comparison of the number of localized N-glycopeptide spectral matches (glycoPSMs), N-glycans, and intact N-glycopeptides from human plasma, urine, cells, and tissues.

Furthermore, we analyzed and compared the number of localized N-glycoPSMs, intact N-glycopeptides, and N-glycan compositions from four clinical samples (i.e., plasma from PCa patients, urine from IgAN patients, HepG2 cells, and tissues from TC patients) for all methods (Figure 3 and Supplementary Table S5). The average numbers of localized N-GlycoPSMs of four clinical samples for each method are summarized and presented in Figures 3A–D. The results clearly show that EThcD-sceHCD is the best (*p *< 0.01, ANOVA), followed by sceHCD, and the other three methods are equal in terms of the number of localized N-GlycoPSMs. Besides, the sceHCD method can provide quality fragmentation for both peptide and glycan moieties, whereas EThcD-sceHCD can generate more informative spectra including both EThcD products and sceHCD products by virtue of the flexibly alternate fragmentation of the precursor ions using EThcD (2 s) and sceHCD (1 s) mode in a duty cycle (3 s) (Zhang et al., 2021b). In contrast, the N-GlycoPSMs of EThcD, HCD-pd-ETD, and sceHCD-pd-ETD scans were much fewer than those of sceHCD or EThcD-sceHCD scans due to the less MS/MS acquisition resulting from the slower scan rate. Then, we compared the average numbers of N-glycan compositions and intact N-glycopeptides. Interestingly, for plasma sample, EThcD-sceHCD can outperform sceHCD (*p *< 0.001, *t*-test) and other methods (*p *< 0.001, *t*-test) in terms of the number of identified N-glycan compositions and intact N-glycopeptides (Figures 3E, I), while for urine, cell, and tissue samples, both EThcD-sceHCD and sceHCD obtain good peptide and glycan sequence coverage with comparable identification number of N-glycan compositions and intact N-glycopeptides (Figures 3F–H,J–L). This may be attributed to the higher complexity of plasma sample, especially the presence of numerous high-abundance proteins, which is not conducive to sceHCD and requires the improvement of fragmentation method for deeper coverage of intact N-glycopeptides particularly from low-abundance proteins. The above results indicated that EThcD-sceHCD has high scanning speed to generate sufficient number of spectra with superior fragmentation quality. Hence, it outperforms other methods in the balance of depth and accuracy of intact N-glycopeptide identification.

**FIGURE 4:**
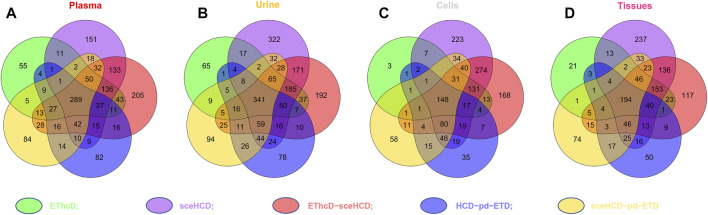
Comparison of the intact N-glycopeptides identified by different tandem mass spectrometry (MS/MS) fragmentation modes.

To evaluate the types of intact N-glycopeptides identified by different methods, we analyzed intact glycopeptides from four clinical samples. As shown in Figure 4, a total of 1,549, 1949, 1,394, and 1,325 intact N-glycopeptides were identified from plasma, urine, cells, and tissues, respectively. However, only 289, 341, 148, and 194 intact N-glycopeptides were commonly identified by all five methods. Of note, sceHCD and EThcD-sceHCD can identify more unique intact N-glycopeptides. These results suggest good complementarity of the five methods, and combinations of different methods (e.g., the combination of sceHCD and EThcd-sceHCD) for the same sample analysis will greatly increase the depth of intact N-glycopeptide identification.

**FIGURE 5:**
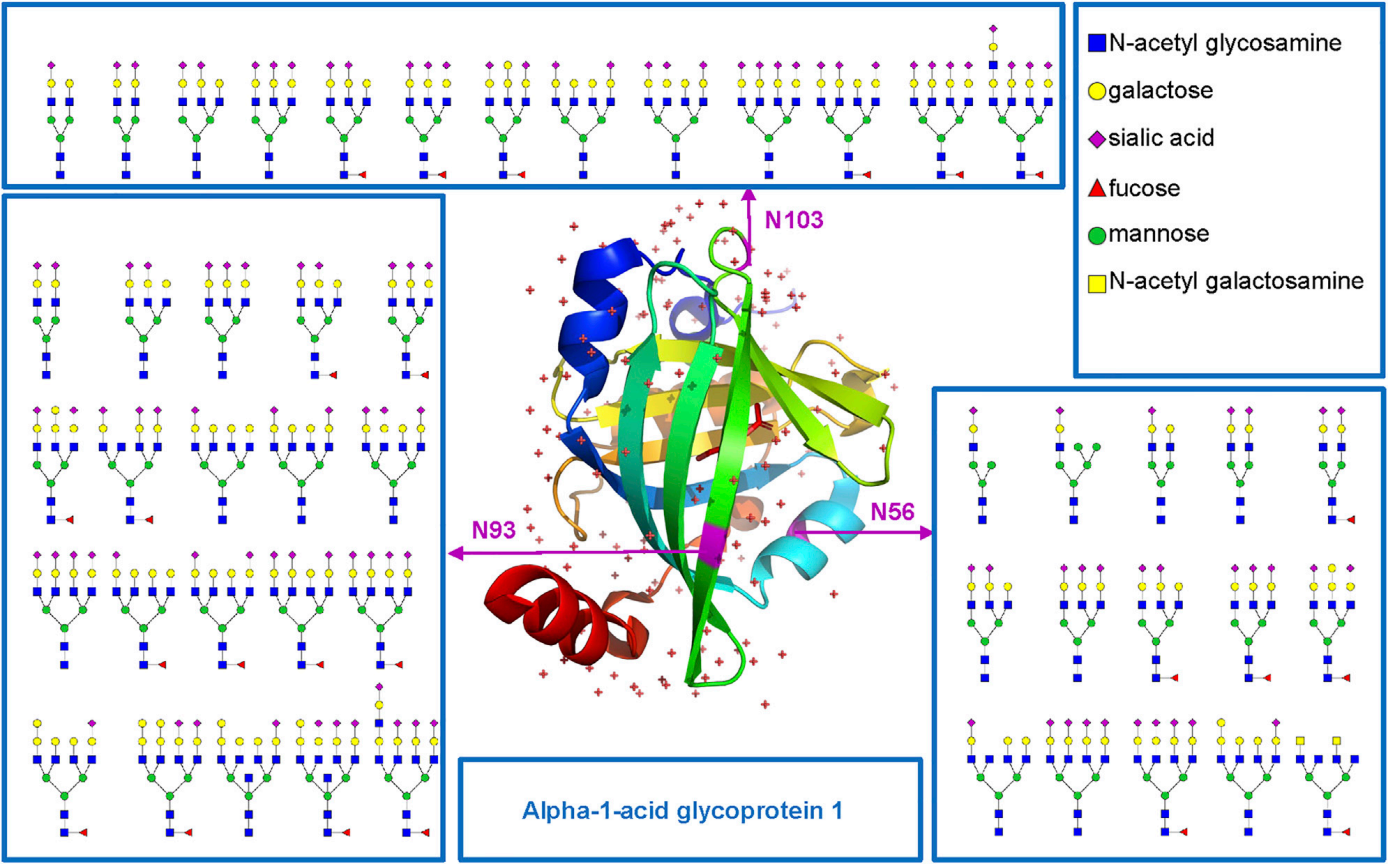
N-glycosites (N56, N93, and N103) and deduced N-glycans were mapped in the three-dimensional structure of the alpha-1-acid glycoprotein 1 (PDB code: 3KQ0) from human plasma.

Based on the comprehensive and multilevel glycoproteomic information of varied clinical samples obtained in this work, we further constructed the glycoproteome databases of clinical samples, which may be potentially translated into clinical applications and provide important reference information for fundamental research. To illustrate the microheterogeneity and macroheterogeneity of glycoproteins, we chose alpha-1-acid glycoprotein 1 to illustrate because it has been used as a standard N-glycoprotein at a lot of glycoprotein studies. In this study, we also found that alpha-1-acid glycoprotein 1 in PCa patients’ plasma is a highly glycosylated protein (Figure 5). As a transport protein in the blood stream, it can bind various ligands and synthetic drugs, and then regulate their distribution and accumulation in the body (Fitos et al., 2006; Zsila and Iwao, 2007). Herein, three N-glycosites (N56, N93, and N103) and 48 intact N-glycopeptides were identified using the five methods (Supplementary Table S6). Specifically, N56, N93, and N103 were located at the α-helix, β-sheet, and loop of the protein and were modified with 15, 20, and 13 N-glycans, respectively. Nearly all N-glycans were sialylated complex type, and half of them were fucosylated. In addition, all the three N-glycosites were commonly modified by eight N-glycans [HexNAc(5)Hex(6)NeuAc(3), HexNAc(4)Hex(5)NeuAc(2), HexNAc(6)Hex(7)NeuAc(4), HexNAc(5)Hex(6)NeuAc(2), HexNAc(6)Hex(7)Fuc(1)NeuAc(4), HexNAc(5)Hex(6)Fuc(1)NeuAc(2), HexNAc(5)Hex(7)Fuc(1)NeuAc(2), HexNAc(5)Hex(6)Fuc(1)NeuAc(3)]. This result suggests that our methods can decipher the microheterogeneity and macroheterogeneity of glycoproteins from complex clinical samples. It is worth noting that five N-glycosites of alpha-1-acid glycoprotein have been found in some studies (Lee et al., 2016; Bollineni et al., 2018; Hwang et al., 2020; Keser et al., 2021; Virág et al., 2021). However, these studies mainly focus on the analysis of N-glycosylation of a recombinant expressed or purified or especially treated alpha-1-acid glycoprotein. To cover all N-glycosites of alpha-1-acid glycoprotein from complex plasma sample, special sample processing (high protein abundance removal, antibodies and affinity chromatography, acid precipitation, multienzyme digestion, etc.) or other mass-spectrometric techniques may also be required.

## Conclusion

This study, to the best of our knowledge, first conducted the comprehensive comparison among EThcD, sceHCD, EThcD-sceHCD-, HCD-pd-ETD, and sceHCD-pd-ETD in the intact N-glycopeptides analyses of clinical samples (plasma, urine, cells, and tissues). Based on the systematical comparison results, we conclude that EThcD-sceHCD is more suitable for the intact N-glycopeptides analysis of clinical samples because it outperforms other methods in the balance of depth and accuracy of intact N-glycopeptide identification. The combination of sceHCD and EThcD-sceHCD will greatly increase the depth of intact N-glycopeptide identification due to their good complementarity, offering more flexible choices for selecting a suitable fragmentation method in glycoproteomic study. These findings would drive glycoproteomic methodological development, guide the software development, and promote clinical application research.

## Data Availability

The datasets presented in this study can be found in online repositories. The names of the repository/repositories and accession number(s) can be found below: http://www.proteomexchange.org/, PXD030288.
